# Supplementary material for: Co-Expression Analysis of Airway Epithelial Transcriptome in Asthma Patients with Eosinophilic vs. Non-Eosinophilic Airway Infiltration
Source: Int J Mol Sci. 2023 Feb 14;24(4):3789. doi: 10.3390/ijms24043789 (PMC9959255; doi:10.3390/ijms24043789)

Co-expression analysis of airway epithelial transcriptome in asthma patients with eosinophilic vs. non-eosinophilic airway infiltration

Pawel Kozlik-Siwiec[1], Sylwia Buregwa-Czuma[2], Izabela Zawlik[3], Aleksander Myszka[3], Joanna Zuk[1], Andzelika Siwiec-Kozlik[1], Jacek Zarychta[1,4], Krzysztof Okon[5], Lech Zareba[2], Jerzy Soja[1], Michał Kępski[2], Jan G. Bazan[2*], Stanislawa Bazan-Socha[1*]

Supplementary material

Supplementary Table S1. Biological processes associated with genes differentially co-expressed between eosinophilic and non-eosinophilic asthma in bronchial brush biopsy samples.
Gene ontology derived from GO Biological Process 2018 by Gene Ontology Consortium.
Note: adjusted p-values >0.05.

**Biological process**	**Gene ontologu accession number**	**Genes**	**p-value**	**z-score**	**combined log(p-value)*z-score**
Rho protein signal transduction	GO:0007266	*EPS8L1 RHOBTB2*	0.00591	-39.98	89.1
regulation of T cell tolerance induction	GO:0002664	*CLC*	0.00956	-239.9	484
negative regulation of myeloid cell apoptotic process	GO:0033033	*MAEA*	0.00956	-239.9	484
regulation of cytokine production involved in immune response	GO:0002718	*CLC*	0.00956	-239.9	484
hemostasis	GO:0007599	*GPI*	0.01115	-205.6	401
folic acid transport	GO:0015884	*SLC19A1*	0.01115	-205.6	401
positive regulation of protein localization to Cajal body	GO:1904871	*CCT7*	0.01273	-179.9	341
regulation of protein localization to Cajal body	GO:1904869	*CCT7*	0.01273	-179.9	341
regulation of T cell mediated immunity	GO:0002709	*CLC*	0.01273	-179.9	341
positive regulation of establishment of protein localization to telomere	GO:1904851	*CCT7*	0.01431	-159.9	295
regulation of immunoglobulin secretion	GO:0051023	*GPI*	0.01431	-159.9	295
positive regulation of immunoglobulin secretion	GO:0051024	*GPI*	0.01431	-159.9	295
potassium ion import across plasma membrane	GO:1990573	*ATP1B1*	0.01431	-159.9	295
thyroid hormone generation	GO:0006590	*DIO3*	0.01589	-143.9	259

regulation of establishment of protein localization to telomere	GO:0070203	*CCT7*	0.01589	-143.9	259
cardiac muscle cell membrane repolarization	GO:0099622	*ATP1B1*	0.01746	-130.8	230
membrane repolarization during action potential	GO:0086011	*ATP1B1*	0.01746	-130.8	230
import across plasma membrane	GO:0098739	*ATP1B1*	0.01746	-130.8	230
relaxation of muscle	GO:0090075	*ATP1B1*	0.01746	-130.8	230
positive regulation of sodium ion transmembrane transporter activity	GO:2000651	*ATP1B1*	0.01746	-130.8	230
relaxation of cardiac muscle	GO:0055119	*ATP1B1*	0.01746	-130.8	230
positive regulation of protein localization to chromosome, telomeric region	GO:1904816	*CCT7*	0.01746	-130.8	230
positive regulation of potassium ion transmembrane transporter activity	GO:1901018	*ATP1B1*	0.01904	-119.9	206
positive regulation of establishment of protein localization	GO:1904951	*CCT7*	0.01904	-119.9	206
positive regulation of ruffle assembly	GO:1900029	*EPS8L1*	0.01904	-119.9	206
sodium ion export	GO:0071436	*ATP1B1*	0.02061	-110.7	187
cellular potassium ion homeostasis	GO:0030007	*ATP1B1*	0.02061	-110.7	187
sodium ion export from cell	GO:0036376	*ATP1B1*	0.02061	-110.7	187
regulation of T cell cytokine production	GO:0002724	*CLC*	0.02061	-110.7	187
membrane repolarization	GO:0086009	*ATP1B1*	0.02061	-110.7	187
transmembrane transport	GO:0055085	*STOML3* *ATP1B1* *SLC19A1*	0.02215	-11.42	18.9
modified amino acid transport	GO:0072337	*SLC19A1*	0.02218	-102.8	170
cell communication by electrical coupling involved in cardiac conduction	GO:0086064	*ATP1B1*	0.02218	-102.8	170
clathrin coat assembly	GO:0048268	*EPS15*	0.02218	-102.8	170
establishment or maintenance of actin cytoskeleton polarity	GO:0030950	*RHOBTB2*	0.02218	-102.8	170
positive regulation of telomerase RNA localization to Cajal body	GO:1904874	*CCT7*	0.02374	-95.94	156
amide transport	GO:0042886	*SLC19A1*	0.02374	-95.94	156

cofactor transport	GO:0051181	*SLC19A1*	0.02374	-95.94	156
cellular sodium ion homeostasis	GO:0006883	*ATP1B1*	0.02374	-95.94	156
negative regulation of RNA metabolic process	GO:0051253	*RPS13*	0.02374	-95.94	156
membrane repolarization during cardiac muscle cell action potential	GO:0086013	*ATP1B1*	0.02374	-95.94	156
positive regulation of potassium ion transmembrane transport	GO:1901381	*ATP1B1*	0.02374	-95.94	156
protein stabilization	GO:0050821	*CCT7* *ATP1B1*	0.02452	-18.94	30.5
cellular monovalent inorganic cation homeostasis	GO:0030004	*ATP1B1*	0.02687	-84.65	133
positive regulation of calcium ion transmembrane transporter activity	GO:1901021	*ATP1B1*	0.02687	-84.65	133
viral entry into host cell	GO:0046718	*EPS15*	0.02687	-84.65	133
regulation of cellular response to growth factor stimulus	GO:0090287	*FBN3*	0.02842	-79.95	124
negative regulation of RNA splicing	GO:0033119	*RPS13*	0.02842	-79.95	124
regulation of telomerase RNA localization to Cajal body	GO:1904872	*CCT7*	0.02842	-79.95	124
drug transport	GO:0015893	*SLC19A1*	0.02998	-75.74	115
folic acid metabolic process	GO:0046655	*SLC19A1*	0.02998	-75.74	115
positive regulation of immunoglobulin production	GO:0002639	*GPI*	0.02998	-75.74	115
Golgi to endosome transport	GO:0006895	*EPS15*	0.03153	-71.96	108
regulation of ruffle assembly	GO:1900027	*EPS8L1*	0.03308	-68.53	101
regulation of cardiac muscle contraction by calcium ion signaling	GO:0010882	*ATP1B1*	0.03308	-68.53	101
vitamin transport	GO:0051180	*SLC19A1*	0.03618	-62.57	90.2
folic acid-containing compound metabolic process	GO:0006760	*SLC19A1*	0.03618	-62.57	90.2
positive regulation of ion transmembrane transporter activity	GO:0032414	*ATP1B1*	0.03618	-62.57	90.2
glycolytic process through glucose-6-phosphate	GO:0061620	*GPI*	0.03926	-57.56	80.9
canonical glycolysis	GO:0061621	*GPI*	0.03926	-57.56	80.9
regulation of potassium ion transmembrane transporter activity	GO:1901016	*ATP1B1*	0.03926	-57.56	80.9
potassium ion homeostasis	GO:0055075	*ATP1B1*	0.03926	-57.56	80.9

glucose catabolic process to pyruvate	GO:0061718	*GPI*	0.03926	-57.56	80.9
sodium ion homeostasis	GO:0055078	*ATP1B1*	0.0408	-55.35	76.9
endocytic recycling	GO:0032456	*EPS15*	0.04234	-53.3	73.2
regulation of actin polymerization or depolymerization	GO:0008064	*EPS8L1*	0.04387	-51.4	69.8
cardiac muscle cell action potential	GO:0086001	*ATP1B1*	0.04541	-49.62	66.6
dicarboxylic acid transport	GO:0006835	*SLC19A1*	0.04541	-49.62	66.6
regulation of activated T cell proliferation	GO:0046006	*CLC*	0.04541	-49.62	66.6
regulation of protein polymerization	GO:0032271	*EPS8L1*	0.04694	-47.97	63.7
ion transmembrane transport	GO:0034220	*STOML3* *ATP1B1*	0.04775	-13.14	17.4
Ras protein signal transduction	GO:0007265	*EPS8L1* *RHOBTB2*	0.04932	-12.91	16.9
positive regulation of telomere maintenance via telomerase	GO:0032212	*CCT7*	0.04999	-44.97	58.5
purine ribonucleoside triphosphate metabolic process	GO:0009205	*ATP1B1*	0.04999	-44.97	58.5

Supplementary Table S2. Molecular functions associated with genes differentially co-expressed between eosinophilic and non-eosinophilic asthma in bronchial brush biopsy samples. Gene ontology derived from GO Molecular Function 2018 by Gene Ontology Consortium. Note: adjusted p-values >0.05.

**Molecular function**	**Gene ontology accession number**	**Genes**	**p-value**	**z-score**	**combined log(p-value)*z-score**
T cell receptor binding	GO:0042608	*EPS8L1*	0.00956	-239.85	484.4
small ribosomal subunit rRNA binding	GO:0070181	*RPS13*	0.01115	-205.59	401.5
signal recognition particle binding	GO:0005047	*SRPRB*	0.01273	-179.89	340.9
armadillo repeat domain binding	GO:0070016	*STRN4*	0.01431	-159.9	294.9
potassium-transporting ATPase activity	GO:0008556	*ATP1B1*	0.01746	-130.83	230
sodium:potassium-exchanging ATPase activity	GO:0005391	*ATP1B1*	0.01746	-130.83	230
sodium ion binding	GO:0031402	*ATP1B1*	0.01904	-119.93	206.3
metalloendopeptidase inhibitor activity	GO:0008191	*RECK*	0.01904	-119.93	206.3
potassium ion binding	GO:0030955	*ATP1B1*	0.01904	-119.93	206.3
Rac guanyl-nucleotide exchange factor activity	GO:0030676	*EPS8L1*	0.02218	-102.79	170
alkali metal ion binding	GO:0031420	*ATP1B1*	0.0253	-89.945	143.6
MHC class II protein complex binding	GO:0023026	*ATP1B1*	0.0253	-89.945	143.6
cysteine-type endopeptidase activity involved in apoptotic process	GO:0097153	*CLC*	0.0253	-89.945	143.6
MHC protein complex binding	GO:0023023	*ATP1B1*	0.02998	-75.743	115.4
lysophospholipase activity	GO:0004622	*CLC*	0.02998	-75.743	115.4
protein phosphatase 2A binding	GO:0051721	*STRN4*	0.03153	-71.956	108
mRNA 5'-UTR binding	GO:0048027	*RPS13*	0.03463	-65.414	95.5
ATPase activator activity	GO:0001671	*ATP1B1*	0.03618	-62.57	90.2

Supplementary Table S3. Signaling pathways associated with transcription factors from the regulatory network of genes differentially co-expressed between eosinophilic and non-eosinophilic asthma in bronchial brush biopsy samples.

Signaling pathway	Associated transcription factors	p-value	q-value	z-score	combined log(p-value) *z-score
Regulation of nuclear SMAD2/3 signaling	CEBPB; HDAC1; GATA3; NR3C1; FOXO3; SIN3B; HNF4A; MYC; EP300; E2F4; E2F5; SMAD2; JUN; SMAD4; SMAD3; VDR; TFE3; ESR1; RUNX2; RUNX1; AR; NCOR1; CREB1; TFDP1; SP1; SP3; TCF3; ATF3	8.73E-37	1.82E-34	-83.78	3021.193231
AP-1 transcription factor network	EGR1; JUN; NR3C1; GATA2; ETS1; HIF1A; ESR1; FOSL1; CREB1; CCND1; SP1; MYC; EP300; CTNNB1; TP53; ATF3	2.86E-18	2.99E-16	-56.20	986.0307212
E2F transcription factor network	HDAC1; TFE3; BRCA1; HIC1; YY1; TFDP1; TRIM28; SP1; MYC; E2F1; EP300; MYBL2; HBP1; E2F4; E2F5; E2F6	6.00E-18	4.18E-16	-53.86	927.6012899
Notch-mediated HES/HEY network	HDAC1; STAT3; ARNT; GATA4; RBPJ; HIF1A; GATA1; RUNX2; YY1; AR; NCOR1; E2F1; EP300; TCF3	1.09E-17	5.70E-16	-70.69	1199.092417
Validated targets of C-MYC transcriptional repression	SMAD2; SMAD4; SPI1; SMAD3; NFYA; HDAC1; NFYC; BRCA1; FOXO3; CREB1; CCND1; SP1; MYC; EP300	7.48E-16	3.13E-14	-53.86	814.7221182
Glucocorticoid receptor regulatory network	EGR1; JUN; SPI1; HDAC1; GATA3; NR3C1; RELA; NFKB1; PBX1; SMARCA4; NR4A1; CREB1; IRF1; EP300; TP53	1.51E-15	5.27E-14	-44.34	657.0726358
Regulation of nuclear beta catenin signaling and target gene transcription	JUN; HDAC1; LEF1; MITF; KLF4; SMARCA4; AR; CCND1; MYC; SALL4; EP300; CTNNB1; HBP1; TCF4	2.18E-14	6.51E-13	-42.95	586.7956942
Regulation of retinoblastoma protein	CEBPB; JUN; SPI1; HDAC1; MITF; RUNX2; SMARCA4; TFDP1; CCND1; E2F1; EP300; PPARG; E2F4	2.92E-14	7.62E-13	-49.23	666.3598742

Regulation of Telomerase	JUN; SMAD3; HDAC1; ESR1; NFKB1; CCND1; SIN3B; WT1; SP1; MYC; IRF1; SP3; E2F1	5.50E-14	1.28E-12	-47.03	623.584993
FOXA1 transcription factor network	FOXA1; AR; CEBPB; JUN; NFIA; SP1; NFIC; EP300; BRCA1; ESR1; FOXA2	2.48E-13	5.18E-12	-60.59	763.8201229
C-MYB transcription factor network	CEBPB; SPI1; NCOR1; CCND1; TRIM28; SP1; MYC; LEF1; EP300; GATA3; ETS1; GATA1	1.77E-11	3.36E-10	-35.47	381.3941217
Validated nuclear estrogen receptor alpha network	CEBPB; JUN; SMAD4; NCOR1; CCND1; HDAC1; MYC; EP300; BRCA1; ESR1; ESR2	2.04E-11	3.55E-10	-41.66	445.3469976
Signaling events mediated by HDAC Class I	YY1; NCOR1; SIN3B; HDAC1; STAT3; EP300; PPARG; GATA2; GATA1; RELA; NFKB1	2.90E-11	4.66E-10	-40.40	425.6891794
HIF-1-alpha transcription factor network	JUN; SMAD4; CREB1; SMAD3; SP1; HNF4A; EP300; ARNT; GATA2; ETS1; HIF1A	2.90E-11	4.33E-10	-40.40	425.6891794
Direct p53 effectors	FOXA1; JUN; NFYA; VDR; NFYC; HIC1; SMARCA4; TFDP1; SP1; E2F1; EP300; TP53; TP63; ATF3	4.65E-11	6.48E-10	-24.95	257.7948324
Regulation of Androgen receptor activity	AR; EGR1; JUN; HDAC1; EP300; SRY; NR3C1; NR2C2; GATA2	1.35E-09	1.76E-08	-41.95	372.1071412
Signaling mediated by p38-alpha and p38-beta	ATF1; CEBPB; JUN; CREB1; MITF; HBP1; ESR1; TP53	2.19E-09	2.70E-08	-51.03	441.8400253
p73 transcription factor network	YAP1; WT1; SP1; MYC; HSF1; EP300; FOXO3; GATA1; TP63; RELA	3.39E-09	3.94E-08	-31.07	263.1731164
Calcineurin-regulated NFAT-dependent transcription in lymphocytes	FOSL1; EGR1; JUN; IRF4; E2F1; PPARG; GATA3; FOXP3	1.10E-08	1.21E-07	-42.15	335.5324137
RXR and RAR heterodimerization with other nuclear receptor	SREBF1; NR4A1; VDR; RARA; PPARG; PPARD	7.93E-08	8.29E-07	-60.59	430.2696264
Notch signaling pathway	YY1; NCOR1; CCND1; HDAC1; MYC; EP300; GATA3; RBPJ	8.39E-08	8.35E-07	-32.86	232.5624802

FOXA2 and FOXA3 transcription factor networks	FOXA1; CEBPB; CREB1; SP1; HNF4A; NR3C1; FOXA2	2.09E-07	1.99E-06	-37.70	251.8464888
Presenilin action in Notch and Wnt signaling	JUN; CCND1; HDAC1; MYC; CTNNB1; RBPJ; PPARD	2.45E-07	2.22E-06	-36.88	243.8556084
HIF-2-alpha transcription factor network	SP1; EP300; ARNT; ETS1; ELK1; POU5F1	7.32E-07	6.38E-06	-42.77	262.4281795
Validated transcriptional targets of AP1 family members Fra1 and Fra2	FOSL1; JUN; CCND1; SP1; EP300; USF2	8.77E-07	7.33E-06	-41.55	251.6791655
ATF-2 transcription factor network	JUN; CREB1; CCND1; EP300; BRCA1; ESR1; ATF3	1.25E-06	1.00E-05	-29.25	172.697571
FOXM1 transcription factor network	CCND1; SP1; MYC; EP300; FOXM1; ESR1	1.99E-06	1.54E-05	-36.36	207.2522483
Validated transcriptional targets of deltaNp63 isoforms	YAP1; VDR; HBP1; POU2F2; TP63; RUNX1	4.07E-06	3.03E-05	-32.32	174.2178406
IL6-mediated signaling events	CEBPB; JUN; MYC; IRF1; STAT3; MITF	4.64E-06	3.34E-05	-31.61	168.6197375
Validated transcriptional targets of TAp63 isoforms	TFAP2C; VDR; SP1; EP300; HBP1; TP63	1.08E-05	7.50E-05	-27.44	136.3231851
Signaling events mediated by HDAC Class II	BCOR; NR3C1; GATA2; GATA1; ESR1	1.63E-05	1.10E-04	-35.64	170.6272481
IL2 signaling events mediated by PI3K	MYC; E2F1; FOXO3; RELA; NFKB1	1.89E-05	1.23E-04	-34.63	163.549873
BCR signaling pathway	JUN; POU2F2; ETS1; ELK1; RELA; NFKB1	3.22E-05	2.04E-04	-22.72	102.0670923
Integrin-linked kinase signaling	JUN; ZEB1; CREB1; CCND1; CTNNB1	6.59E-05	4.05E-04	-26.93	112.6035437
LKB1 signaling events	SMAD4; CREB1; MYC; ESR1; TP53	8.15E-05	4.86E-04	-25.78	105.4357932
Validated targets of C-MYC transcriptional activation	FOSL1; SMAD4; SMAD3; MYC; EP300; TP53	1.06E-04	6.17E-04	-18.41	73.15299554

Retinoic acid receptors-mediated signaling	HDAC1; VDR; RARA; EP300	1.35E-04	7.63E-04	-34.63	133.9714725
TGF-beta receptor signaling	YAP1; SMAD2; SMAD4; SMAD3; CTNNB1	1.59E-04	8.75E-04	-22.44	85.23980904
CD40/CD40L signaling	JUN; MYC; RELA; NFKB1	1.78E-04	9.55E-04	-32.32	121.156723
Coregulation of Androgen receptor activity	AR; CCND1; CTNNB1; TCF4; BRCA1	2.42E-04	0.001267	-20.54	74.26029048
IL4-mediated signaling events	CEBPB; SPI1; IRF4; SP1; ETS1	2.63E-04	0.001338	-20.20	72.32655319
Trk receptor signaling mediated by PI3K and PLC-gamma	EGR1; CREB1; CCND1; FOXO3	3.66E-04	0.001823	-26.93	92.53414197
Downstream signaling in naive CD8+ T cells	EOMES; FOSL1; EGR1; JUN; ELK1	4.10E-04	0.001994	-18.36	62.1904905
ErbB1 downstream signaling	ATF1; EGR1; JUN; CREB1; STAT3; ELK1	5.02E-04	0.002383	-13.85	45.70010227
Regulation of cytoplasmic and nuclear SMAD2/3 signaling	SMAD2; SMAD4; SMAD3	5.20E-04	0.002416	-42.77	140.4574345
IFN-gamma pathway	CEBPB; IRF1; STAT3; EP300	5.52E-04	0.002508	-24.24	78.96896859
Hypoxic and oxygen homeostasis regulation of HIF-1-alpha	ARNT; HIF1A; TP53	6.20E-04	0.002757	-40.40	129.57832
Angiopoietin receptor Tie2-mediated signaling	ETS1; ELK1; RELA; NFKB1	0.001108	0.004823	-20.20	59.69672704
Signaling events mediated by HDAC Class III	EP300; FOXO3; TP53	0.001868	0.007967	-27.97	76.31116825
p53 pathway	YY1; TRIM28; EP300; TP53	0.002105	0.008797	-17.01	45.53025547

Supplementary Table S4. Impact of kinase perturbations in animal studies on genes differentially expressed between eosinophilic and non-eosinophilic asthma in bronchial brush biopsy samples. Kinase perturbation studies data retrieved from Gene Expression Omnibus using Enrichr software. Only terms with p-value <0.05 are shown; p-values adjusted by Fisher's exact test >0.05.

**Affected kinase and study type**	**GEO accession number**	**Affected genes**	**p-value**	**z-score**	**combined log(p-value) *z-score**
		Genes up-regulated due to kinase perturbation			
HIPK1 (knockout)	GSE39253	*GPI*<br>*MAEA*<br>*STRN4*	0.0120	-14.39	27.63348
TGFBR2 (knockout)	GSE22989	*MRPL14*<br>*CCT7*<br>*EPS15*	0.0120	-14.39	27.63348
		Genes down-regulated due to kinase perturbation			
IKBKE (siRNA-mediated knockdown)	GSE27869	*C14ORF159*<br>*RAPH1*<br>*ASB3*	0.0120	-14.39	27.63348

Supplementary Table S5. Cell-type enrichment analysis of histone ChIP-seq peak marks of human cell lines annotated within the Encyclopedia of DNA Elements (ENCODE).

Note: adjusted p-values >0.05.

Histone modification and cell line	p-value	Affected genes
H3K9ac fibroblast of lung hg19	0.035726	ASB3; RADX; CCT7; MRPL14; PPP2R3B; RPS13; SLC19A1
H3K27me3 fibroblast of lung hg19	0.045305	CLC; RADX; DIO3; EPS8L1; FADS6; FBN3; SLC19A1; SRCRB4D; STOML3
H3K36me3 myocyte mm9	0.054552	ASB3;RPS13
H3K27me3 SK-N-SH hg19	0.122431	FBN3;FADS6;EPS8L1;DIO3;CLC;RADX
H3K9ac K562 hg19	0.166164	FADS6;RAPH1;MRPL14;PPP2R3B;CCT7; ATP1B1;SRCRB4D;SLC19A1
H3K4me1 G1E mm9	0.209147	FADS6;RAPH1;EPS8L1;SRPRB;SRCRB4D; RECK
H3K9me3 A549 hg19	0.211366	CABP5;ANKRD26P1;TTC3P1;DIO3; ATP1B1
H3K4me1 CD14-positive monocyte hg19	0.211366	GPI;FADS6;EPS8L1;CCT7;RECK
H2AFZ skeletal muscle myoblast hg19	0.211366	FADS6;RAPH1;ASB3;SLC19A1;RHOBTB2
H3K9ac mammary epithelial cell hg19	0.211366	EPS8L1;MRPL14;PPP2R3B;ASB3;RHOBTB2
H3K27me3 T-cell acute lymphoblastic leukemia hg19	0.211366	GPI;STOML3;EPS8L1;SRCRB4D;STRN4
H3K27ac HepG2 hg19	0.211366	GPI;MAEA;RADX;CCT7;RPS13
H3K27ac GM12878 hg19	0.211366	RAPH1;PPP2R3B;CCT7;ATP1B1;SLC19A1
H3K4me3 skeletal muscle myoblast hg19	0.211366	MRPL14;PPP2R3B;RADX;CCT7;RPS13
H3K27ac astrocyte hg19	0.211366	C14ORF159;ASB3;STRN4;RPS13;RHOBTB2
H3K27me3 myotube hg19	0.211366	FBN3;CABP5;EPS8L1;CLC;SRCRB4D
H3K9me1 keratinocyte hg19	0.211366	GPI;MAEA;CCT7;RECK;STRN4
H3K9ac CD14-positive monocyte hg19	0.211366	RAPH1;EPS8L1;MRPL14;PPP2R3B;CCT7

Supplementary Table S6. Curated resources used in gene set enrichment analysis and construction of interaction networks.

Interactome	Description
Libraries used for gene set enrichment analysis	
ENCODE histone modifications	Database of histone modification probed by ChIP-seq, ChIP-chip and qChIP studies[123]
GO Biological process	Library of gene associated with biological programs accomplished by multiple molecular activities[124,125]
GO molecular function	Library of genes associated with molecular-level activities[124,125]
PID	The Pathway Interaction Database collection of curated and peer-reviewed pathways composed of human molecular signaling and regulatory events and key cellular processes[126]
Interactomes used for network construction	
ENCODE	Transcription factor and gene target data derived from the ENCODE ChIP-seq data[127,128]
JASPAR	Transcription factor targets derived from the JASPAR TF binding site profile database[129]
ChEA	Transcription factor targets database inferred from integrating literature curated Chip-X data[130]
TF-miRNA coregulatory interactions	RegNetwork repository[131]

Supplementary Figure S1. Gene regulatory network – transcription factor-gene interactions (JASPAR). Transcription factor targets database inferred from integrating literature curated JASPAR data.

Green edges link to TFs targeting only DCGs up-regulated in EA, orange edges to TFs targeting DCGs upregulated in both EA and NEA, red edges – TFs targeting only DCGs upregulated in NEA.

Figure prepared using Cytoscape v. 3.7.1[132], processed using Inkscape Project (202) Inkscape v. 1.0.2.

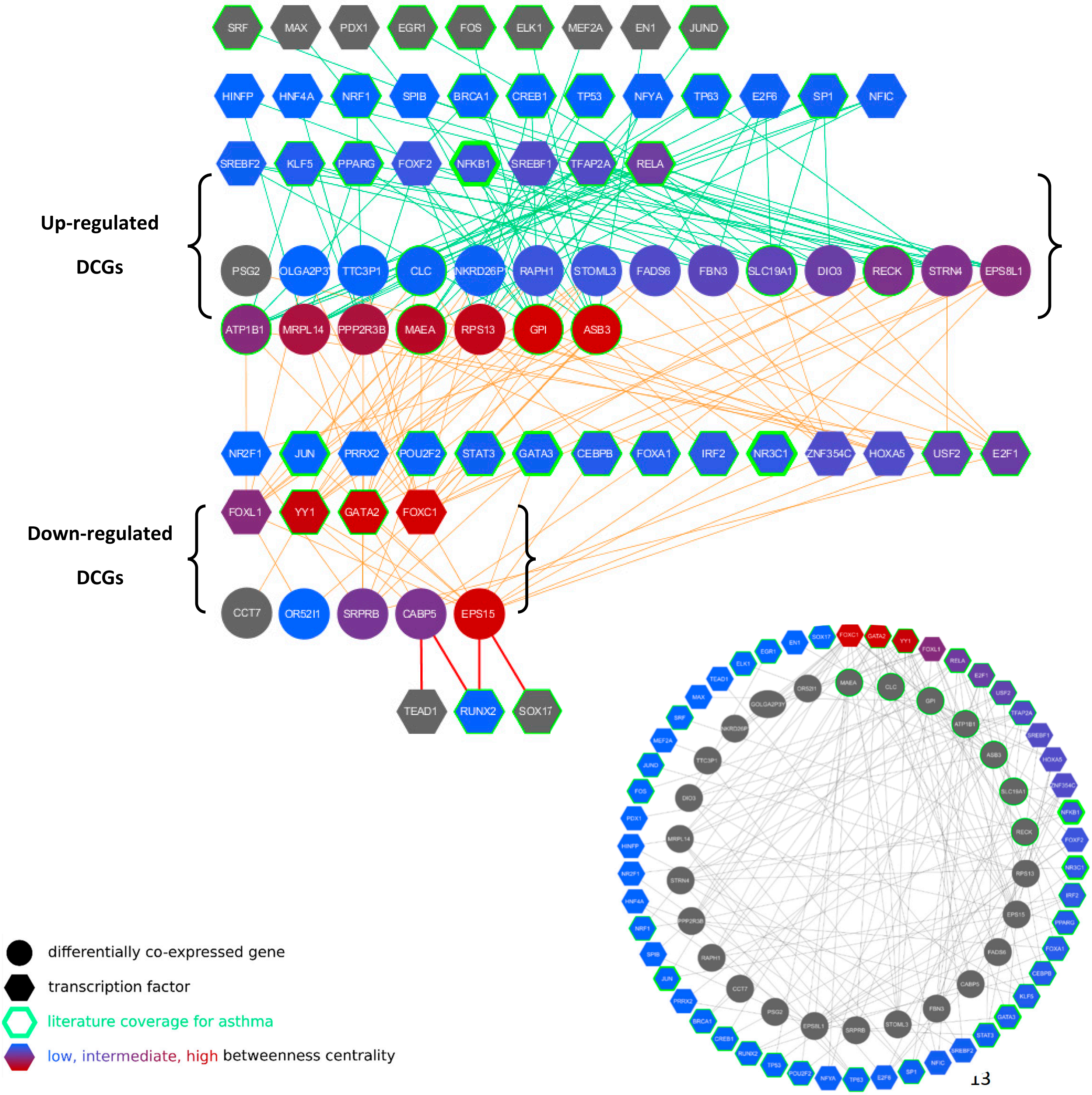

Supplementary Figure S2. Gene regulatory network – transcription factor-gene interactions (Chip-X).

Transcription factor targets inferred from Chip-X curated database.

Figure prepared using Cytoscape v. 3.7.1[132], processed using Inkscape Project (202) Inkscape v. 1.0.2.

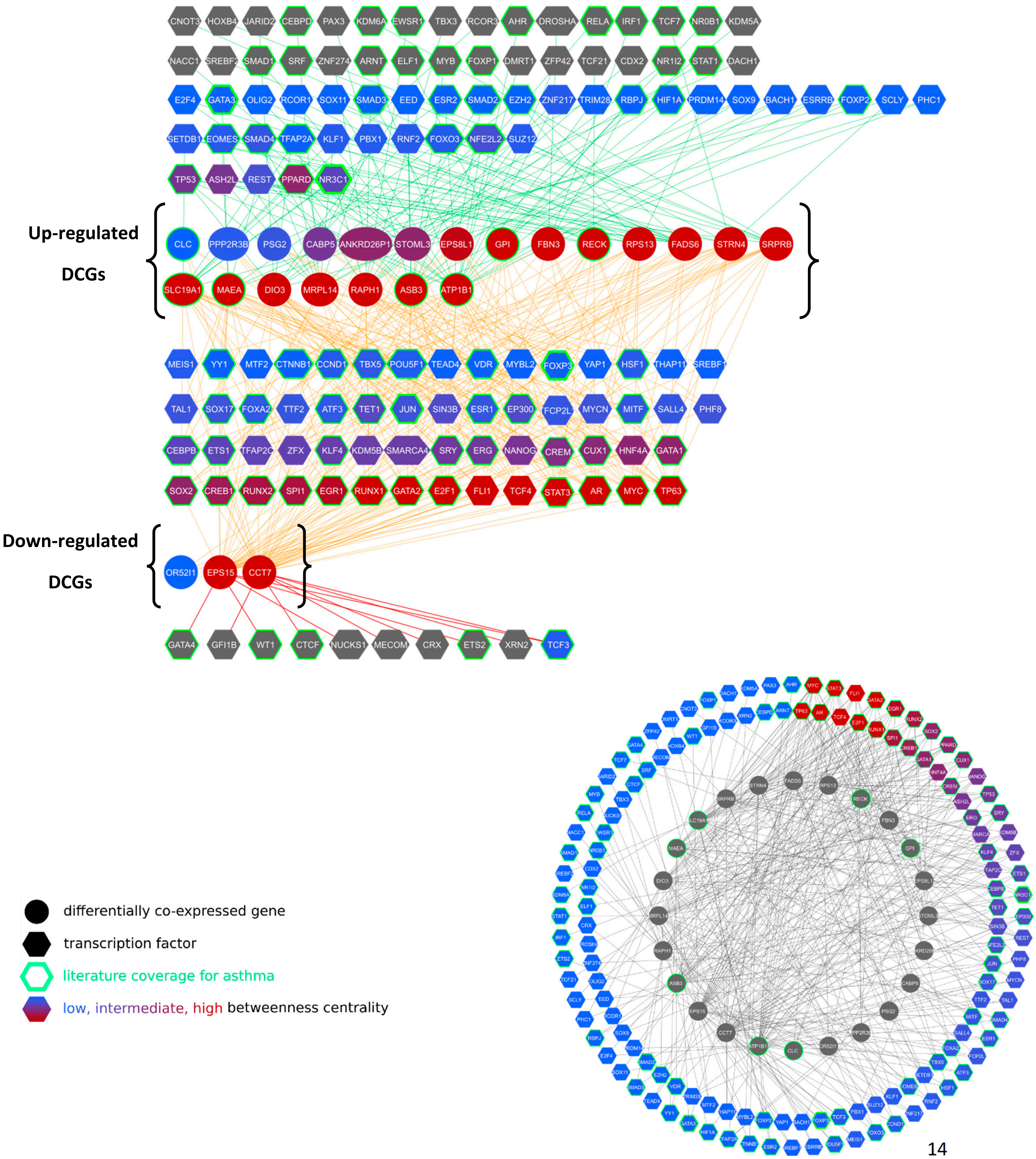

Supplementary Figure S3. Gene regulatory network – transcription factor-gene interactions (ChIP-seq).

Transcription factor and gene target data derived from the ENCODE ChIP-seq data. Peak intensity signal <500, predicted regulatory potential score <1 (BETA minus algorithm).

Figure prepared using Cytoscape v. 3.7.1[132], processed using Inkscape Project (202) Inkscape v. 1.0.2.

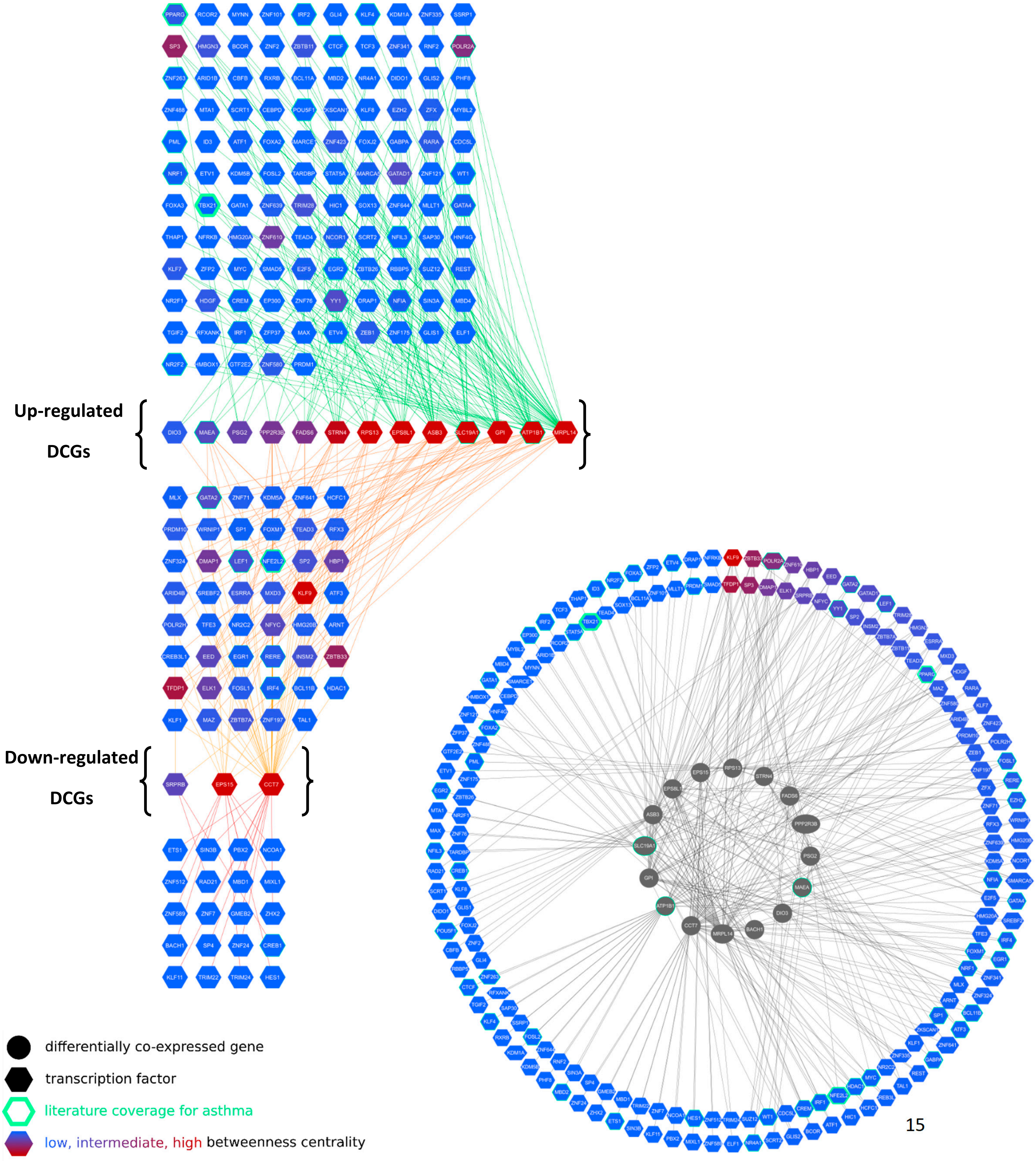

Supplement: Supplementary file 1 [file ijms-24-03789-s001.zip › ijms-2112296-supplementary.pdf]
